# Nutritional status and dietary habits in older adults with fixed implant dental prostheses: a case-control study

**DOI:** 10.3389/fnut.2024.1373372

**Published:** 2024-09-26

**Authors:** George Homsi, Mats Trulsson, Anastasios Grigoriadis, Abhishek Kumar

**Affiliations:** ^1^Division of Oral Diagnostics and Rehabilitation, Department of Dental Medicine, Karolinska Institutet, Huddinge, Sweden; ^2^Tandvården Sergel, Praktikertjänst, Stockholm, Sweden; ^3^Academic Center for Geriatric Dentistry, Stockholm, Sweden

**Keywords:** mini nutritional assessment, oral functions, dietary records, malnutrition, mastication, food choices

## Abstract

**Aim:**

To evaluate the nutritional status, nutritional risk, and dietary habits of patients treated with bimaxillary implant-supported fixed prostheses in comparison with a group of natural dentate patients.

**Methods:**

A study group (*n* = 25, 8 women, mean age = 70.6 ± 7.5 years) with bimaxillary implant-supported fixed prostheses and a control group (*n* = 25, 13 women, mean age = 69.0 ± 5.3) with a mean of 27.7 ± 1.8 natural teeth were recruited. The nutritional status and nutritional risk of the participants were evaluated with Mini Nutritional Assessment (MNA) and Seniors in the Community: Risk Evaluation for Eating and Nutrition; (SCREEN-14), while the dietary habits were recorded by data from a three-day dietary record. The data were analyzed with the Mann–Whitney U-test and independent *t*-test to evaluate the differences between the groups.

**Results:**

The results showed that although both the groups had normal nutrition status as revealed by the MNA scores the study group showed significantly higher BMI (*p* = 0.005) but lower SCREEN-14 (*p* = 0.012) scores, than the control group. The results also showed that higher SCREEN-14 scores were significantly associated with higher odds of being in the control group, with an odds ratio of 1.159 (*p* = 0.024). Further, the results of the analysis of the dietary records showed that the participants in the study group consumed fewer meals (*p* = 0.006) and fewer varieties of food (*p* < 0.001), particularly fewer fruits (*p* = 0.011) than the control group.

**Conclusion:**

The results indicate that people with fixed implant prostheses may be susceptible to nutritional deficiencies according to the SCREEN-14 scores compared to their natural dentate counterparts. Further, people with implant prostheses also tend to have higher BMI and consume a smaller variety of foods, especially fruits, than the natural dentate control group.

## Introduction

Oral health and oral function significantly influence nutritional status and overall quality of life, particularly in the aging population (1–4). The oral function is significantly affected by deteriorating dental status, fewer teeth, especially posterior teeth that occlude, and the type of dental prosthesis. The reduced ability to chew, as well as swallowing difficulties due to decreased saliva secretion, may lead to poor food choices, negatively affecting nutrition (5, 6). Studies have suggested that diet and dietary behaviors play an integral role in the prevention of common diseases such as diabetes cardiovascular disease and cognitive decline (7–9). However, the relationship between oral status/function and dietary choices, dietary habits, and nutritional status is often neglected in routine clinical practice (9–11).

Prosthodontic rehabilitation procedures are generally believed to enhance masticatory function and improve oral health-related quality of life (12). However, the effect of prosthodontic rehabilitation (alone) on nutritional status remains a topic of ongoing debate (13). Studies have shown that replacing removable dentures with fixed implant-supported dentures can lead to better masticatory function and increased consumption of fibrous, nutrient-rich foods (14, 15). Additionally, two-implant overdentures have been associated with significantly healthier participants, as indicated by improvements in anthropometric measures and blood nutrient levels (16). Despite these findings, other research indicates that while fixed implant prostheses may enhance masticatory function and improve food selection compared to removable prostheses, they do not always result in improved nutritional intake or status (17, 18). A recent systematic review confirmed that although individuals with fixed implant-supported prostheses experience significant improvements in masticatory function compared to those with conventional removable prostheses, this improvement does not necessarily lead to better nutrient bioavailability (19). It has been suggested that dental implant patients in general have a good health status, which is necessary for the surgical procedures involved in implant placement (19). Furthermore, chewing function is only one of many factors influencing nutritional status (1). Beyond chewing ability, factors like sensorimotor regulation, food choices, systemic diseases, dietary restrictions, and educational and socioeconomic factors play crucial roles in overall health and nutritional well-being (1, 20). Therefore, it is important to investigate the intricate relationship between different dental interventions and overall nutritional well-being.

It has previously been reported that patients with bimaxillary implant-supported fixed prostheses show signs of masticatory impairment despite receiving satisfactory and well-functioning prostheses (21). We have also reported that this group, despite exhibiting signs of masticatory impairments, does not self-report any limitation of jaw function or compromised oral health-related quality of life (22). It has been suggested that chewing function is one of the important physiological contributors to overall general health including the nutritional status (1, 9). Therefore, we believe that people with bimaxillary implant-supported prostheses who show signs of poor masticatory performance (21, 22) may be susceptible to nutritional deficiencies. Hence, the current study aims to evaluate the nutritional status, nutritional risk, and dietary behavior in a group of patients treated with bimaxillary implant-supported fixed prostheses in comparison to a control group of people with natural teeth. We specifically hypothesized that the implant patient group would exhibit signs of poor nutritional status and increased nutritional risk, which would be reflected in their Mini Nutritional Assessment (MNA) scores, Body Mass Index (BMI), and Seniors in the Community: Risk Evaluation for Eating and Nutrition (SCREEN-14) scores. Additionally, we anticipated that this group would display altered dietary habits compared to the control group.

## Materials and methods

The current case-control study follows the recommendations of the Declaration of Helsinki II and was independently reviewed and approved by the Regional Ethical Review Board, Stockholm, Sweden with the reference number Dnr 2018/1963-31. All participants were given information about the main objectives of the study and the method of data collection before their participation. All participants from both groups gave their verbal and written informed consent before participation.

### Study participants

The current study includes the dataset from a previous study that assessed masticatory function in patients with bimaxillary implant-supported prostheses (22). Accordingly, both subjective and objective evaluations were conducted to comprehensively understand the impact of these prostheses on oral function. It was evident that patients with bimaxillary implant-supported prostheses show poor masticatory performance (21) and there was no agreement in the objective and subjective measures of mastication (22). Accordingly, the sample size was performed using G*Power version 3.1.9.7 to determine the number of participants required for testing the study hypothesis. The results indicated that a sample of approximately 52 participants would be needed to achieve 60% power in detecting a medium effect size, with a significance criterion of *α* = 0.05. Given the constraints of using existing data, the sample size in the current study reflects the number of available participants who met the inclusion criteria rather than a new sample size calculation. Therefore, no separate sample size was calculated.

Thus, the study group in the current study included 25 (age = 70.6 ± 7.5 years, including 8 women and 17 men) edentulous participants who have been rehabilitated with bimaxillary implant-supported fixed prostheses. The participants in the study were found through a database search engine at the Global Health Partner (GHP), Specialisttandläkarna, Nacka, Sweden. Participants treated with bimaxillary implant-supported fixed prostheses at least 1 year before they participated in the study were screened. Those scheduled for routine follow-up within the next 2 years were included in the study. The participants in the study group were contacted by one of the researchers (GH) who initiated the first contact with eligible candidates via phone. During this call, he explained the study’s objectives. Candidates who expressed satisfaction with their treatment and agreed to participate were sent a written consent form and other details by mail. In general, the study group reported that they were satisfied with their prostheses and had no obvious complaints (21). The primary inclusion criteria for the study group were that the participants must have been using their prostheses for a minimum of 1 year and had no evident complaints regarding their prostheses. This ensured that the prostheses had been adequately tested over time and that any immediate issues had likely been resolved. None of the participants reported any major limitation of jaw function or compromised oral health-related quality of life, as reported previously (22). During the rehabilitation procedure, a standardized method combining a metal framework and acrylic teeth replacing 12 teeth in each arch in a mutually protected occlusion, was used. All the prostheses were constructed on an abutment level attached to Straumann^®^ or Lifecore^®^ implants. A typical two-stage methodology with a healing period of 3–6 months after the installation of the implants was applied. Further, no clinical signs of mucositis such as swelling and/or bleeding on probing, or any progressive bone destruction could be detected around any of the dental implants.

The control group (*n* = 25, mean ± standard deviation age = 69.0 ± 5.3, including 13 women and 12 men) included people with natural dentition. Patients scheduled for routine dental checkups at the Tandvården Sergel, Praktikertjänst, Stockholm, Sweden were contacted. Those accepted to participate in the study were provided with the informed consent forms and other details via mail. Participants for inclusion in the control group were selected based on the following criteria: they did not possess any tooth-supported or implant-supported prostheses, nor did they have removable partial dentures. Additionally, they did not report any immediate need for dental treatment at the time of their inclusion in the study. This ensured that the control group comprised individuals with relatively healthy oral conditions and no urgent dental issues, allowing for a clear comparison with the study group. Accordingly, the participants in the control group on average had 27.7 ± 1.8 number of natural teeth. A chairside clinical examination was conducted to determine if there were any obvious defects or problems related to biting or chewing. Additionally, no participant (in any of the groups) reported symptoms or signs of orofacial pain, temporomandibular disorders, systemic diseases, or neurological disorders of the masticatory system.

### Nutritional assessment

The nutritional status and nutritional risk of all participants were assessed using MNA and SCREEN-14 (previously known as SCREEN-II) nutritional assessment tools. The two questions-based screening tools were sent to the participants via post a minimum of 5 weeks before the clinical session. Additionally, the participants were also asked to maintain a three-day dietary record. During the clinical assessment, participants had the opportunity to clarify any uncertainties or ask questions about the study.

### MNA^®^

The nutritional status was assessed using the MNA^®^ questionnaire (23, 24). MNA^®^ is a validated nutritional assessment tool developed through a collaborative research program in several countries to rapidly evaluate nutritional status among older patients in homes, clinics, hospitals, and nursing homes (23, 24). The Swedish version of the questionnaire is composed of simple measurements and brief questions that can be completed in less than 10 min. The MNA includes different components such as anthropometric measurements, global assessment, dietary questionnaire, and other subjective assessments. The anthropometric measurements (including weight, height, and weight loss) provide valuable insights into changes in body composition and weight loss. The global assessment considers lifestyle, medication, and mobility, identifying malnutrition risk factors such as living alone, taking multiple medications, or having pressure sores. The dietary questionnaire, adapted for elderly individuals across different countries, assesses meal frequency, food and fluid intake, and autonomy in feeding. Finally, the subjective assessment is based on self-perception of health and nutrition and serves as a good indicator for evaluating nutritional status. In this study, only anthropometric measurements (Body Mass Index) and global assessment were used; it is recommended that people with an MNA score of 12 or greater do not require further intervention, scores of 8–11 indicate risk of malnutrition, and a score of 7 or less indicates malnutrition. The BMI scores were categorized as follows: underweight (BMI < 18.5), normal weight (BMI 18.5–24.9), overweight (BMI 25–29.9), and obesity (BMI ≥ 30).

### SCREEN-14

The nutritional risk was assessed with the SCREEN-14 questionnaire, a valid and reliable tool for the identification of risk for impaired nutritional status in community-living older adults (25). SCREEN-14 has been developed using both psychometric and clinometric methods. The Swedish version (26) comprises 14 questions/items with several sub-questions and provides information regarding weight loss, food intake, and risk factors for malnutrition. The items have different ranges, with a total score in the range of 0–64 can be obtained. A score of <50 indicates a high nutritional risk while 50–53 indicates moderate nutritional risk and >53 is a low risk.

### Dietary records

A three-day dietary record was used to evaluate the dietary habits based on the food preferences of the two groups (27, 28). The dietary records were prospective and open-ended and included food records of two weekdays and a weekend, (i.e., Saturday or Sunday). The participants in both groups were specifically instructed to note down the time of meal and everything they ate or drank during the 3 days. The purpose was to determine whether the two groups differed in their dietary habits. The time of the meal was an indicator of an individual meal. Hence, the total number of meals during the 3 days was counted and compared between the groups. The solid food intake, i.e., food items other than the fluids was categorized into six food groups based on a food preference questionnaire designed for adolescents and adults (29). The six food groups were vegetables, fruits, meat/fish, dairy, snacks, and starches. The total food items (excluding the fluids) were counted for each participant and the percentage of each food group was calculated.

### Statistical analysis

The data are presented as mean ± standard deviation and percentages. The data were analyzed with the IBM Statistical Package for Social Sciences (Version 25.0. Armonk, NY: IBM Corp.) statistical software program. The normality distribution was checked by histogram plots and the Shapiro–Wilk test. All normally distributed data such as diet categories (meat/fish, dairy, and starches) were analyzed with student *t*-tests (parametric) to compare the two groups. All skewed data such as the MNA, and SCREEN-14 scores were analyzed with the non-parametric Mann–Whitney U-test. Binomial logistic regression was performed to investigate the association between groups (Study and Control) as the dependent variable and indicators of nutrition (MNA scores, BMI, and SCREEN-14 scores) as explanatory variables. We calculated odds ratios (ORs) along with their 95% confidence intervals (CIs). These ORs help predict how independent variables influence the odds of dependent variables.

## Results

All participants completed both the SCREEN-14 and MNA^®^ questionnaires except for two individuals in the study group who did not complete the dietary records. The mean and the standard deviations of all the measured variables are also summarized in Table 1.

**Table 1:** Mean and standard deviation of the measured variables in the study group and control group.

	Study group	Control group
MNA score	13.3 ± 1.3	12.9 ± 1.1
BMI	27.6 ± 4.1	24.7 ± 4.2
SCREEN-14	48.5 ± 6.1	52.7 ± 4.5

MNA, Mini Nutritional Assessment; BMI, Body Mass Index.

SCREEN-14, Seniors in the Community: Risk Evaluation for Eating and Nutrition.

### MNA^®^

The results showed that two participants (8%) in the study group and one (4%) participant in the control group were at risk of malnutrition. The mean MNA scores showed that in general both the groups had normal nutritional status.

The mean BMI scores for the study group and control group are presented in Table 1. It was also observed that none of the participants in any of the groups were underweight. However, eight (32%) of the participants in the study group and five (20%) in the control group were overweight. Besides, eight (32%) in the study group and three (12%) in the control group had a BMI score of ≥30, indicating obesity. The results of the statistical analysis showed that the study group presented significantly higher BMI scores than the control group (*U* = 168.5, *p* = 0.005). In other words, the study group participants were overweight, while the control group participants were normally weighted according to their BMI status.

### SCREEN-14

The SCREEN-14 scores showed that fourteen (56%) participants in the study group and five (20%) participants in the control group were at a high risk of malnutrition. Besides, three (12%) participants in the study and eight (32%) in the control group showed a moderate risk of malnutrition. Overall, the mean SCREEN-14 score in the study group was 48.5 ± 6.1 indicating a high risk of malnutrition, compared to 52.7 ± 4.5 indicating a moderate risk in the control group. Also, there was a significant difference in the SCREEN-14 scores between the two groups (*U* = 183, *p* = 0.012) (Table 1).

### Dietary records

**Figure 1:**
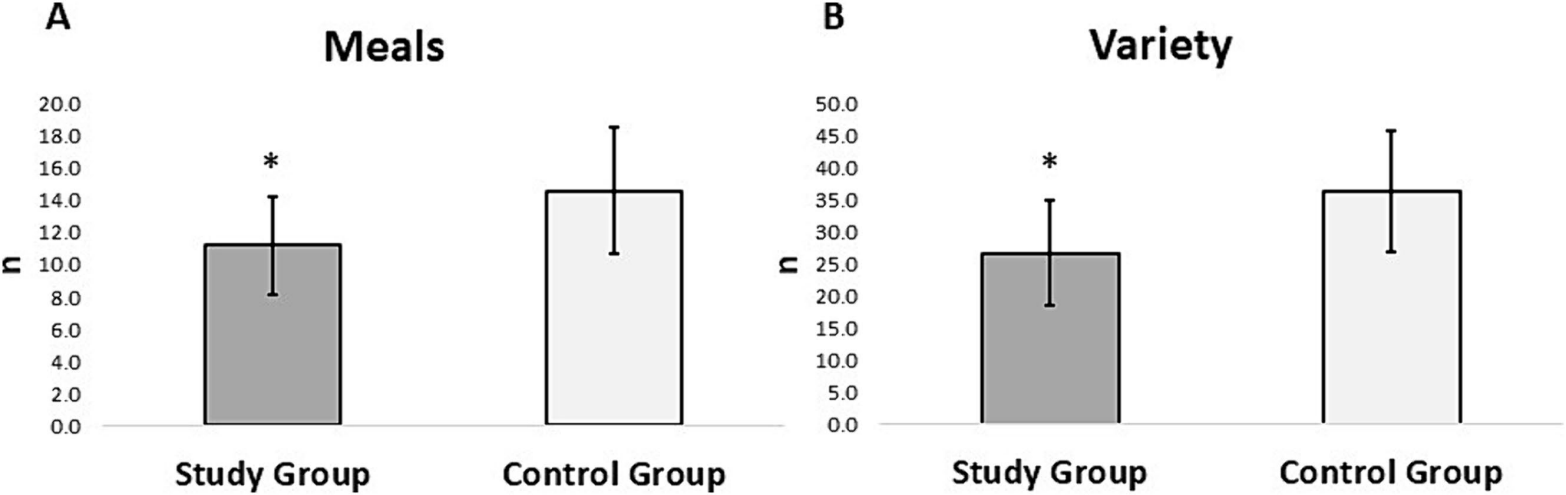
Mean and standard deviation of **(A)** number of meals and **(B)** variety of food consumed by the participants in the study and control group from the three-day diet records. The asterisk (_*_) denotes significant differences between the groups.

**Figure 2:**
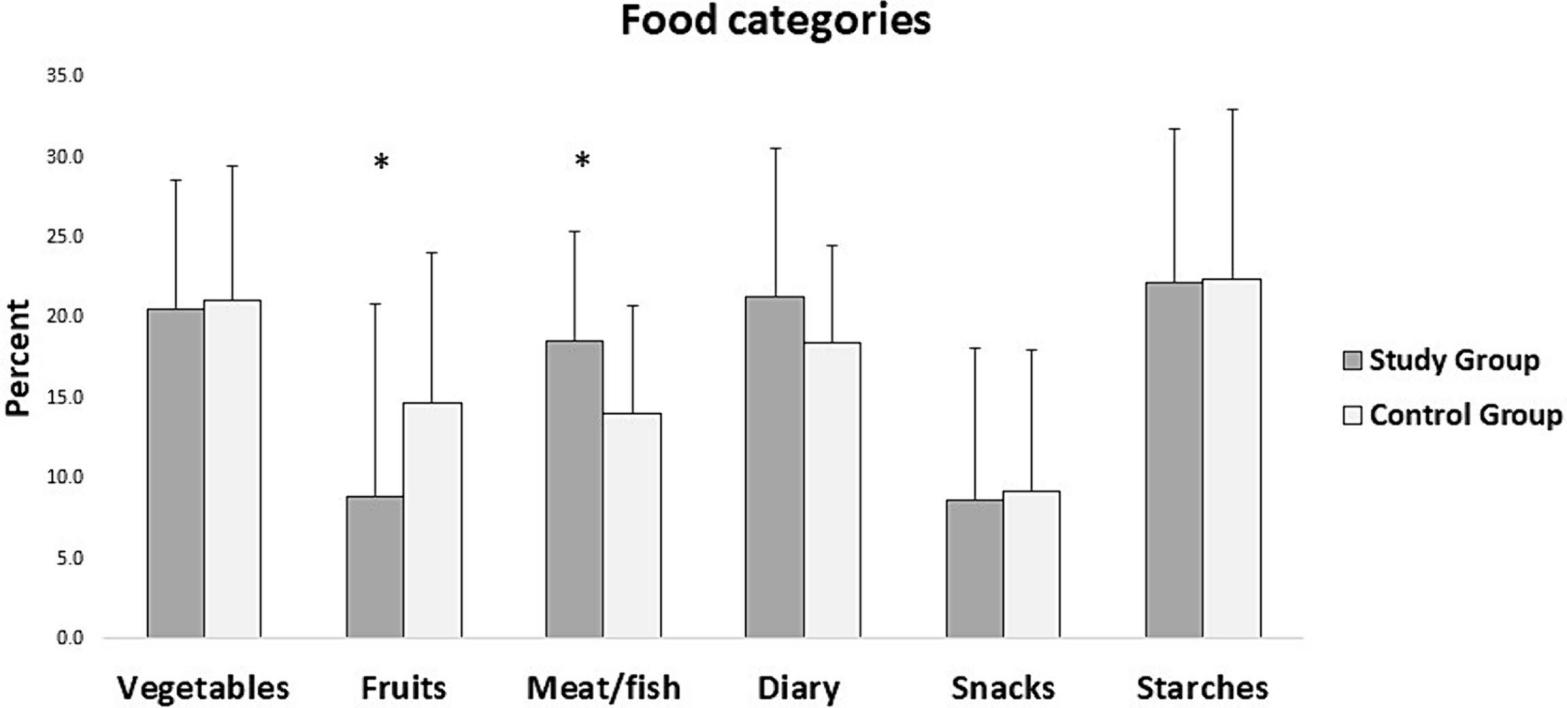
Mean and standard deviation of different constituents of food consumed by the participants in the study and control group from the three-day diet records. The asterisk (_*_) denotes significant differences between the groups.

The three-day dietary records from the participants in both groups were individually evaluated. The results showed that the participants in the study group during the 3 days significantly consumed fewer number of meals (*p* = 0.006) (Figure 1A) and a smaller variety of food (*p* < 0.001) than the control group (Figure 1B). The number of food items consumed during the 3 days was categorized into six food groups based on the food preference questionnaire (29) and the percentage of each category was calculated. The percent food items for each food group category were then compared between the groups. The results showed that the study group consumed significantly more meat/fish than the control group (*p* = 0.025). The results also showed that the study group consumed a significantly lower number of fruits than the control group (*p* = 0.011) (Figure 2). However, there were no differences in the consumption of vegetables (*p* = 0.918), dairy (*p* = 0.274), snacks (*p* = 0.715), or starches (*p* = 0.931) between the groups (Figure 2).

### Association between dental status and nutritional indicators

Binomial logistic regression was conducted to examine the effect of MNA score, BMI, and SCREEN-14 scores on group membership (study vs. control group). The results of the analysis showed that the overall model was statistically significant (*χ*^2^(3) =11.924, *p* = 0.008). The model explained 28.8% (Nagelkerke R^2^) of the variance in the outcome and correctly classified 73.5% of cases. The results also showed that the regression coefficients for the MNA score (*p* = 0.224) and BMI (*p* = 0.165) were not statistically significant. However, the regression coefficient for the SCREEN-14 score was statistically significant (*p* = 0.024). The odds ratio (Exp(B)) for SCREEN-14 was 1.159, 95% CI [1.019, 1.318], indicating that higher SCREEN-14 scores (better nutritional status) were associated with higher odds of being in the control group. In other words, SCREEN-14 scores were a significant predictor of being in the control group, with an odds ratio of 1.159. The power of the study was calculated based on the observed odds ratio (OR) of 1.159, a significance level (*α*) of 0.05, and a sample size of 50. The *post hoc* power of the study was approximately 74%.

## Discussion

The current study evaluated the nutritional status of people with bimaxillary implant-supported prostheses and compared it with a group of people with natural dentition. The results of the study showed significantly higher MNA, BMI, and lower SCREEN-14 scores in the study group than in the control group. The odds ratio for SCREEN-14 was 1.159, 95% CI [1.019, 1.318], indicating that higher SCREEN-14 scores (better nutritional status) were associated with higher odds of being in the control group. In other words, the finding suggests that better nutritional risk scores are more commonly associated with individuals who have natural dentition compared to those with implant-supported prostheses. The three-day dietary records showed that the study group consumed fewer meals and a smaller variety of foods than the control group. Further, results of the dietary records also showed that the study group consumed significantly more meat/fish but less fruits than the control group. Overall, the SCREEN-14 scores showed that the study group was more susceptible to nutritional deficiencies than the control group. In addition, BMI scores indicated that the study group was overweight compared to the normal weighted control group. Evaluation of the dietary records suggests that people with bimaxillary implant-supported fixed prostheses perhaps do not change their diet despite receiving a fixed implant bridge. It is suggested that additional measures, such as diet counseling and nutritional advice, may be necessary to encourage individuals with implant-supported fixed prostheses to modify their diet and adopt healthier eating habits.

One potential limitation of the current study was the dietary records which are typically subject to bias when completed by the participants. Besides, it requires a degree of motivation and cooperation which if not present can influence the findings and applicability to a larger population. Another potential limitation is that recording foods often tends to affect the choice and quantity of food consumed (30). Consciousness of recording type and amount of food consumed may alter dietary behavior, resulting in “reactivity bias” (31). Since recording measurements such as weighed food records, constituents and amount of the food are time-consuming, the participants were unable to fully provide this information. Therefore, in the current study, we only evaluated the time of meal as an indicator of the number of meals the participants ate and the number of food items as an indicator of the variety of food. Further, the food groups were identified based on the food preference questionnaire although we have not quantified the food preference specifically and only categorized the food consumption based on the categories in the food preference questionnaire. However, it has been suggested that a three-day dietary record is reliable (32) keeping a high degree of motivation without jeopardizing compliance caused by posing too much burden on the participants through a longer period (30, 33, 34). It is also important to recognize that the tools to evaluate nutritional status and nutritional risk (i.e., MNA and SCREEN-14) have certain limitations. While they can still be valuable tools for identifying individuals at risk of malnutrition they cannot be used as part of a comprehensive assessment of an older adult’s health and nutritional status. Further, several factors such as level of education, socioeconomic conditions, marital status, and living situation can influence the nutritional status. While the current study did not record these factors it may be important for future research to consider these as potential confounders to better understand the complex interplay between oral health, socioeconomic factors, and overall well-being. Additionally, given that the current study partially uses the data set from the previous study no separate sample size calculation could be determined and this could be a limitation of the current study. Also, the study may be slightly underpowered. However, although the sample size may be slightly inadequate, the current dataset allows us to detect differences and contributes valuable insights to the existing body of knowledge investigating dental and nutritional health.

Masticatory function plays an important role in food oral processing and is an important contributor to nutritional status and quality of life, especially in older individuals (1–4). The masticatory function is often improved after replacement of lost teeth with suitable dental prostheses (35, 36). Furthermore, it is reported that replacing removable prostheses with fixed prostheses improves masticatory function and increases consumption of protein and fiber-rich foods (37) However, as mentioned above, it is not clear if oral rehabilitation alone can result in better nourishment or influence the dietary behavior and food preferences in older individuals (13, 38). Participants with severely compromised dental status have reported no change in nutritional status despite receiving a combined implant prosthesis with fixed or removable prostheses (39, 40) or implant-supported mandibular overdentures (41). However, prosthetic replacement of lost teeth with bimaxillary implant-supported fixed prostheses is considered a highly advanced method of prosthetic rehabilitation. Whether it (bimaxillary implant-supported fixed prostheses) results in better nourishment, or comparable nourishment to naturally dentate individuals is not clear, and no studies have been performed in this group of patients. Accordingly, our results showed significant differences in the MNA and SCREEN-14 scores between a group of participants rehabilitated with bimaxillary implant-supported prostheses and their age and sex-matched natural dentate counterparts. It can be inferred from the results that participants in the study group with bimaxillary implant-supported prostheses were more susceptible to nutritional deficiencies compared to the control group. Our results are corroborated by another study that compared the nutritional status, dietary intake, and oral health-related quality of life in older people with complete dentures and their natural dentate controls (42). The study found that approximately 21.3% of complete denture wearers were at risk of malnutrition, while none of the natural dentate group faced this risk as assessed by MNA (42). In the current study, despite no significant differences in the MNA scores between the groups, the SCREEN-14 scores indicated that the study group was more susceptible to nutritional deficiencies. Further, those with higher SCREEN-14 scores had significantly greater odds of being in the control group.

The MNA is widely used to evaluate the nutritional status of older adults, focusing on aspects like anthropometric measurements, dietary intake, and overall health status. It categorizes individuals into groups such as normal, at risk of malnutrition, or malnourished based on a cumulative score. In the current study, despite differences in MNA scores between groups, both were classified as normal. This suggests that while there may be numerical variations in MNA scores, they did not cross the threshold to indicate clinically significant differences in nutritional status according to the MNA criteria. On the other hand, SCREEN-14 (Seniors in the Community: Risk Evaluation for Eating and Nutrition, Version II) assesses nutritional risk differently and considers factors such as appetite, food intake, and functional status, potentially identifying individuals at risk of nutritional deficiency earlier or in different circumstances than the MNA or perhaps highlighting subtler distinctions that the MNA does not capture.

As mentioned above, people with bimaxillary implant-supported fixed prostheses tend to perform poorly in the masticatory function test in comparison to natural dentate controls (21, 22). Specifically, the participants in the study group are unable to comminute hard viscoelastic test food into tiny pieces and also show poor mixing ability in two two-color chewing gum mixing tests (21, 22). It is inferred that older people can have chewing problems despite being provided with well-made dental prostheses (21). As particle size is an important determinant of energy and nutrient bioavailability (4, 43–46); people with bimaxillary implant-supported fixed prostheses can also have chewing problems and may be at a higher risk of malnutrition. Although malnutrition has a multifactorial etiology, chewing problems could be contributing to malnutrition associated with aging (1). Hence, it is suggested that the inability to adequately comminute and mix food could influence nourishment and therefore should be evaluated adequately after oral rehabilitation procedures.

All participants in our study, self-reported good general health without a history of chronic systemic disease or a neurological disorder associated with oral functions. Further, the participants in both groups lived independently (not in nursing homes or aged care facilities) although some availed of domestic house-help services. Despite this, the SCREEN-14 scores showed that the study group was at significantly higher risk of malnutrition than the moderate-risk control group. These findings are similar to previous studies from several countries that report the risk of malnutrition in community-dwelling older adults (47–50). It has been specifically reported that 35% of the community-dwelling older adults in Sweden are at moderate risk of malnutrition while 30% are at higher risk (26, 51). While it is not surprising that people living independently are at risk of malnutrition, the fact that those with implant prostheses are at a higher risk may be concerning. Alternatively, poor dietary habits and increased consumption of cariogenic foods may lead to more dental caries, periodontitis, and subsequently teeth loss (52, 53). In the current study, the poor nutritional status of individuals with dental implants may occur as a result of poor dietary habits, and not necessarily due to their dental status. Consequently, in the current study, it can be assumed that the study group probably already had poor dietary habits before losing their natural teeth, affecting their BMI values and nutritional statuses/risks throughout life. People who maintain a fiber-rich and lower carbohydrate diet not only maintain better nutritional status but are also likely to have better oral health (54, 55). It could be that they (the study group) retained their dietary habits even after the oral rehabilitation. Thus, oral rehabilitation alone may not be sufficient to improve a person’s nutritional status, and more interventions such as diet counseling and behavior modification may be required to holistically improve nutrition status (56). Therefore, it is suggested that oral rehabilitation procedures should also aim to restore function and improve nutritional status together with constructing esthetically pleasing and durable prostheses (20, 57–59).

In the current study, it was observed that the two groups differed in their dietary habits. A particular observation was that the study group consumed fewer meals and a smaller variety of foods particularly, fruits, than the control group. People with dental prostheses have shown evidence of impaired chewing function in their ability to perform on par with the natural dentate people, in food comminution and mixing ability tests (21, 22). As a result of impaired chewing performance, they are unable to comminute larger varieties of food items and perhaps restrict themselves from eating the foods that are difficult to comminute. Several studies have suggested that impaired chewing ability is associated with inadequate and poor-quality diet, and malnutrition (60, 61). Consequently, these people are more likely to be susceptible to nutritional deficiencies due to the reduced consumption of fruits (current study) and other micronutrients because of their inability to chew or process certain foods. However, the results also showed that the participants in the study group tended to consume more meat/fish. Studies have suggested that cooking methods can influence the nutritive quality (62, 63) and protein modifications in meat (64). Therefore, it is hypothesized that although people with chewing difficulty, i.e., people with dental implants in the current study eat more meat and fish, but they perhaps overcook the food and, in the process, lose vital nutrients. However, in the current study the cooking methods, the individual constituents of the meals, and the texture of the food could not be evaluated. Yet, it could be hypothesized that people with compromised chewing function tend to eat more processed foods especially foods that are soft and easy to chew, and as a result, are often overweight. Accordingly, in the current study, the study group had significantly higher BMI scores than the control group and it is suggested that this group eats more processed food. Although BMI is not typically gender-specific or age-specific, recent evidence suggests the need to reconsider age-and gender-specific (or race) cutoff values for BMI (65, 66). A comprehensive analysis of dietary habits, including individual meal constituents, nutritive value, and cooking methods, along with specific BMI thresholds for different age groups and genders, would provide further insights. Future research studies should ensure sufficient statistical power and consider potential confounders, including educational and socioeconomic factors.

## Conclusion

The results of the current study preliminarily indicate that people, despite being provided with well-made implant prostheses, may be susceptible to nutritional deficiencies reflected in poor SCREEN-14 scores compared to their natural dentate counterparts and may show signs of poor adaptation to a healthier diet. In addition, people with implant prostheses also tend to have higher BMI and consume a significantly smaller variety of foods especially fruits compared to the natural dentate control group. Thus, it is suggested that oral rehabilitation alone may not be sufficient to improve nutritional status. Multidisciplinary interventions, including diet counseling and behavior modification, may be necessary to achieve a comprehensive improvement in nutritional health (56, 67).

## Data Availability

The raw data supporting the conclusions of this article will be made available by the authors, without undue reservation.
